# Characteristics and Prognosis of a Contemporary Cohort with Myocardial Infarction with Non-Obstructed Coronary Arteries (MINOCA) Presenting Different Patterns of Late Gadolinium Enhancements in Cardiac Magnetic Resonance Imaging

**DOI:** 10.3390/jcm12062266

**Published:** 2023-03-15

**Authors:** Valentina Bucciarelli, Francesco Bianco, Alessia Di Francesco, Piergiusto Vitulli, Annaclara Biasi, Martina Primavera, Sara Belleggia, Giuseppe Ciliberti, Federico Guerra, Jelena Seferovic, Antonio Dello Russo, Sabina Gallina

**Affiliations:** 1 Cardiovascular Sciences Department—AOU “Ospedali Riuniti”, 60126 Ancona, Italy; valentina.bucciarelli@ospedaliriuniti.marche.it; 2 Department of Neurosciences, Imaging and Clinical Sciences, Gabriele d’Annunzio University of Chieti-Pescara, 66100 Chieti, Italy; 3 Cardiology and Arrhythmology Clinic, University Hospital “Umberto I-Lancisi-Salesi”, Marche Polytechnic University, 60123 Ancona, Italy; 4 Cardiovascular Division, Brigham and Women’s Hospital, Harvard Medical School, 75 Francis Street, Boston, MA 02115, USA

**Keywords:** myocardial infarction with non-obstructed coronaries (MINOCA), cardiac magnetic resonance imaging, late gadolinium enhancement patterns, myocarditis

## Abstract

Background: To analyze the characteristics and prognosis of a contemporary cohort of patients with myocardial infarction with non-obstructed coronaries (MINOCA) were referred for cardiac magnetic resonance (CMR) imaging, focusing on late gadolinium enhancement (LGE) patterns. Methods: We retrospectively examined and prospectively followed up with 135 patients (49 ± 21 years old, 48% female) undergoing CMR imaging due to a MINOCA diagnosis from 2014 to 2016. We grouped and analyzed the sample according to ischemic (focal or transmural) and non-ischemic LGE patterns. The primary outcome was cardiac-related death; the secondary outcome was a composite of cardiac-related rehospitalizations, the new occurrence of acute myocardial infarction (AMI), heart failure (HF), or arrhythmias. Results: CMR exams were performed after a median of 28 days from the acute event. One-third of the ischemic MINOCA were first managed as myocarditis, while CMR helped to adopt a different therapy regimen in 22% of patients (30/135). After a median follow-up of 2.3 years, more cardiac-related deaths occurred in the ischemic than non-ischemic group (2 vs. 1, *p* = 0.36), but it was not statistically significant. The ischemic group also experienced more cardiac-related-rehospitalizations (42%, *p* < 0.001). In a multivariable Cox regression model, dyslipidemia, reduced left ventricular ejection fraction, ST-elevation at the hospitalization, and the LGE transmural pattern were the independent predictors of cardiac-related rehospitalizations. Conclusions: In a contemporary cohort of MINOCA patients who underwent CMR, ischemic and non-ischemic patterns had distinct features and outcomes. Among the MINOCA patients, CMR can identify patients at higher risk who require more aggressive therapeutic approached and strict follow-up.

## 1. Introduction

Myocardial infarction with non-obstructive coronary arteries (MINOCA) is conventionally represented by an acute myocardial infarction (AMI) with no angiographic obstructive coronary artery disease: stenosis ≥ 50% diameter in a major coronary epicardial vessel [1].

MINOCA is found in about 5–6% of all patients with acute coronary syndromes referred for coronary angiography, [1,2] with a prevalence of 1–13% in patients presenting with myocardial infarction [3,4,5]. Although the interest in this condition is increasing in recent years, contemporary studies continue to consider MINOCA as a rather heterogeneous group [2,5,6]. The definition of MINOCA is constantly evolving. Previously, “MINOCA” contained all patients who presented with cardiac symptoms (i.e., chest pain) associated with raised myocardial injury blood biomarkers (i.e., troponins) but non-obstructive coronaries on angiography, without necessarily having ruled out non-ischemic causes such as myocarditis [1,2,7].

The recently released 4th Universal Definition of Myocardial Infarction stated that the diagnosis of MINOCA requires an underlying ischemic mechanism [7]. In this context, cardiac magnetic resonance (CMR) imaging, with its multiparametric capabilities in evaluating cardiac structure, function, and tissue characterization, can provide a reliable diagnosis or a reclassification of the presumed diagnosis of myocardial infarction; therefore, it is considered an important diagnostic tool by the European Society of Cardiology working group on myocardial infarction with non-obstructive coronary arteries [6]. CMR imaging could provide good discrimination between patients with ischemic MINOCA from those with non-ischemic MINOCA [8,9]. 

On the other hand, very few studies excluded specific non-ischemic diseases as myocarditis, cardiomyopathies, and Takotsubo syndrome (TTS) [10,11]. The data on the prognosis of these patients are somewhat conflicting, and thus generates results that are often not comparable [12]. Therefore, we hypothesized that among MINOCA patients, a distinction between ischemic and non-ischemic CMR patterns could have significant clinical implications, and we sought to determine the characteristics and prognosis of a contemporary cohort of patients with MINOCA, who underwent CMR, focusing on late gadolinium enhancement (LGE) patterns.

## 2. Materials and Methods

We reviewed 250 medical records of patients admitted to our department due to a MINOCA diagnosis from 2014 to 2016 that underwent a CMR exam; then, we enrolled patients according to our inclusion/exclusion criteria.

Inclusion criteria were acute chest pain at the hospital admission, with new or presumed new significant ST-T changes or new left bundle branch block (LBBB), cardiac troponin rise-and-fall >99th percentile of the upper reference level on serial assessments, and non-obstructive coronary arteries lesions in the angiogram. We excluded all the patients with coronary lesions ≥ 50% (*N* = 20) and patients with a previous history of myocardial infarction (*N* = 45) or cardiomyopathies (*N* = 15). We also excluded participants that ended in a final CMR diagnosis of cardiomyopathy (*N* = 35): dilated (*n* = 10), hypertrophic (*n* = 20), amyloidosis (*n* = 5). As our analysis was specifically focused on LGE patterns, we excluded all patients with a CMR-confirmed TTS diagnosis at the time of enrollment.

A total of 135 patients (49 ± 21 years old, 48% female) constituted the final sample that was followed-up for a median of 2.3 years through outpatient visits in our outpatient department. The data were collected utilizing the available digitally stored medical records; additional phone calls were made for completeness. The primary outcome was cardiac-related-death, and the secondary outcome was a composite of cardiac-related rehospitalizations and the new occurrence of AMI, heart failure (HF), or arrhythmias.

In acquiescence with the Declaration of Helsinki, all enrolled patients gave written informed consent at the time of their evaluation, stating that data and images may be subsequently used for research purposes. Given the retrospective nature of the study, no ethical committee approval was required.

### 2.1. Coronary Angiography and CMR Acquisition Protocols

The angiographic procedures were done from a radial or femoral arterial access, depending on the age of the patients and vascular access availability. All patients received 5000 IU of unfractionated heparin and 200 μg of nitrates before the insertion of the guidewire to control the vasomotor tone. Basal angiographies were obtained in different angiographic views to better visualize and characterize the coronary anatomy.

All the CMR imaging were performed utilizing a 1.5-Tesla (Achieva; Philips Medical System, Best, Netherland) applying a comprehensive protocol including cine, T2-weighted (for myocardial edema), and early and late gadolinium enhancement (LGE) sequences. An intravenous gadolinium–chelate contrast agent (gadobutrol) was administered at a dose of 0.1 mmol/kg^−1^ of body weight. Images were acquired 2–3 min after contrast injection (early gadolinium), whereas LGE images were acquired 10–15 min after contrast injection using a standard inversion recovery segmented gradient echo sequence.

### 2.2. Variables Definition

**Figure 1:**
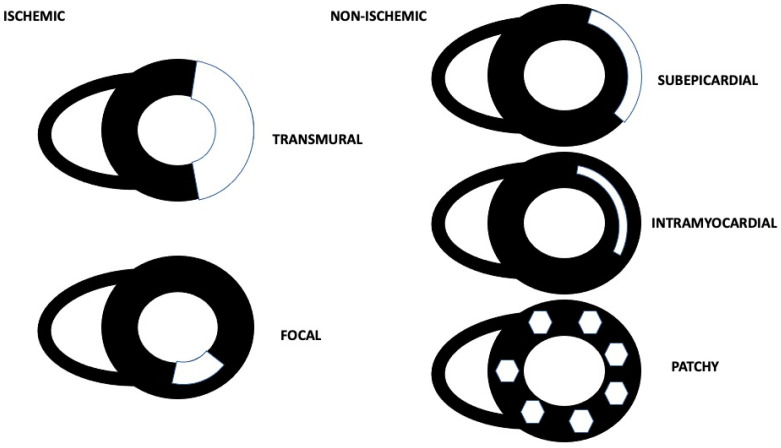
Ischemic and non-ischemic CMR LGE patterns.

For our purposes, we grouped and analyzed the samples according to ischemic (focal or transmural) and non-ischemic LGE patterns (Figure 1). The ischemic pattern was defined as a subendocardial area of enhancement at the CMR sequences acquired after gadolinium contrast agent administration. Non-ischemic patterns were defined as any LGE area presenting an intramyocardial, subepicardial, or patchy enhancement distribution [13].

Regarding the ischemic pattern, a transmural pattern was defined as an area of enhancement involving more than the 70% of the left ventricular wall; conversely, the focal one was defined as a non-transmural, segmental, and restricted area of LGE involving <30% of the left ventricular wall [14]. Myocardial edema was considered present when the ratio of signal intensity between the myocardium and the mean signal intensity of the skeletal muscle was >2 on T2-STIR images [15].

Myocarditis was diagnosed based on fulfilling two of the three Lake Louise Criteria: T2-STIR sequences detecting myocardial edema; early gadolinium sequences detecting hyperemia; or epicardial, intramyocardial (also known as mid-myocardial), or patchy LGE [16]. The early gadolinium enhancement ratio was used for the assessment of hyperemia [14].

TTS was diagnosed based on the T2-STIR images detecting myocardial edema and regional wall motion in the mid-cavity or apical distribution with no myocardial LGE, all in accordance with the modified Mayo Clinic criteria [17,18].

### 2.3. Statistical Analysis

Categorical values are expressed as an absolute number and percentage, while continuous variables as the mean and standard deviation (±SD) or median and interquartile intervals (Q1, Q3), as appropriate.

The comparisons between normally distributed, continuous variables across the LGE group patterns, non-ischemic and ischemic (focal and transmural), were made utilizing the Student’s *t*-test or non-parametric test, while categorical variables were compared by the χ2 test or Fisher’s exact test when appropriate. The variables that were not normally distributed were compared by the Kruskal–Wallis test. The normal distribution was assessed utilizing the Kolmogorov–Smirnov test.

Cumulative event-free survival rates over time were obtained using the Kaplan–Meier method. The log-rank test was used to compare the event-free survival curves. Univariable and multivariable associations of risk covariates with mortality were assessed using Cox proportional hazard regression analyses. Only variables with a *p*-value less than 0.05 at the univariable analysis entered the multivariable model. The latter were age, sex, the presence of comorbidities, laboratory tests at admission, and the different types of CMR patterns discovered at the CMR exams.

All statistical analyses were performed with Stata v14.1 (StataCorp, College Station, TX, USA). *p*-values less than 0.05 were considered statistically significant.

## 3. Results

A CMR exam was performed after a median of 28 days from the acute event. General characteristics, comorbidities and cardiovascular risk factors, laboratory test results, and therapy are presented in Table 1, according to non-ischemic and ischemic (focal and transmural) LGE patterns.

In our cohort of patients, 59% of MINOCA patients had non-ischemic LGE patterns at the CMR exam: *n* = 43 subepicardial, *n* = 21 intramyocardial, and *n* = 16 patchy. One-third of the ischemic MINOCA group were initially managed as myocarditis, while CMR helped to adopt a different regimen of therapy in 22% (30/135) of them, introducing the antiplatelet therapy and statins after CMR.

Ischemic patients were more often female, older, and with a higher prevalence of hypertension, diabetes mellitus, and dyslipidemia than non-ischemic patients. MINOCA patients showing a transmural LGE pattern at the CMR exam had higher incidences of hypertension and diabetes mellitus, had a history of atrial fibrillation, and presented at admission with ST-elevation (Table 2).

After a median follow-up of 2.3 years, more cardiac-related deaths occurred in ischemic than non-ischemic patients (2 vs. 1, *p* = 0.36), but it was not statistically significant. The latter were classified as myocardial infarction in one case, for the ischemic group, and two as sudden cardiac death. The ischemic group experienced more rehospitalizations [42% (23/55) vs. 17% (14/80), *p* < 0.001], HF [25% (14/55) vs. 14% (11/80), *p* < 0.001], and AMI recurrences [13% (7/55) vs. 0% (0/80), *p* < 0.001]; meanwhile, the non-ischemic group experienced more arrhythmias [56% (45/80) vs. 5% (3/55), *p* < 0.001]. The latter were the new onset of non-sustained ventricular tachycardia (*n* = 28), ventricular ectopic beats (*n* = 11), supraventricular/atrial tachycardia (*n* = 4), heart block of varying degrees (*n* = 3), and atrial fibrillation (*n* = 2).

**Figure 2:**
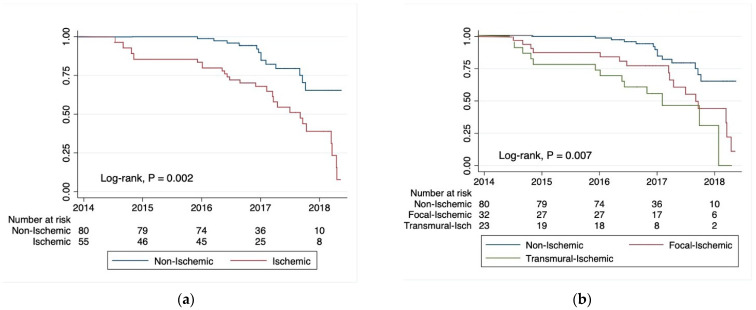
Kaplan–Meier survival estimates for cardiac-related rehospitalizations due to the new occurrence of acute myocardial infarction (AMI), heart failure (HF), or arrhythmias: (**a**) non-ischemic vs. ischemic CMR LGE patterns; (**b**) non-ischemic vs. focal-ischemic and transmural CMR LGE patterns.

The ischemic group presented the worst prognosis (log-rank test: *p* = 0.007), especially in the transmural pattern. On the contrary, the focal and the non-ischemic ones had a better prognosis (log-rank tests: *p* = 0.002) (Figure 2).

In the multivariable Cox regression model, hypercholesterolemia [HR 1.14; 95% CI (1.04, 1.42), *p* < 0.001], ST-elevation at hospitalization [HR 6.26; 95% CI (3.19, 6.68), *p* = 0.001], reduced left ventricular ejection fraction [HR 3.60; 95% CI (1.45, 8.97), *p* = 0.006], and the LGE transmural pattern [HR 6.01; 95% CI (4.90, 7.53), *p* < 0.001] were the independent predictors of the secondary outcome (Table 3).

## 4. Discussion

In our study, we found that MINOCA patients presenting an ischemic CMR pattern were more often female, with more comorbidities and risk factors, including hypertension, diabetes mellitus, and dyslipidemia. One-third of the ischemic MINOCA patients were initially managed as myocarditis, while CMR helped to adopt a different regimen of therapy. More cardiac-related deaths occurred in the ischemic than non-ischemic group, and the ischemic group experienced more rehospitalizations, HF, and AMI recurrences, while the non-ischemic group had more arrhythmias. Accordingly, dyslipidemia, reduced left ventricular ejection fraction (<50%), ST-elevation at hospitalization, and the LGE transmural pattern were found to be independent predictors of rehospitalizations, HF, and AMI recurrences.

These findings support the concepts that MINOCA patients presenting an ischemic LGE pattern at the CMR evaluation have different characteristics and outcomes than non-ischemic ones. [19] In particular, this population seems to have more comorbidities and cardiovascular risk factors. In addition, we registered a gender difference between the etiologies. In particular, women were more prone to have an ischemic MINOCA than men. The converse has been observed in other studies, with men found to be more likely to have a non-ischemic etiology on CMR than women (55% v 41%, *p* < 0.001) [20]. The higher prevalence of the non-ischemic etiology in males is consistent with the epidemiology of myocarditis, which appears to be the most frequent cause of non-ischemic MINOCA [20,21].

To the best of our knowledge, this is the first study focused on the prognostic role of LGE patterns in a population of patients presenting with MINOCA. Moreover, it adds to previous literature that discriminates between MINOCA from an ischemic mechanism of myocardial injury and from a non-ischemic one (mimicking MINOCA). This is of particular interest considering the different therapy regimens that should be adopted in these cases and the uncertainties regarding the optimal medical therapy in MINOCA, particularly the ischemic ones [22,23].

It has been largely demonstrated the role of CMR in establishing a diagnosis or sorting out a previous misdiagnosis of myocardial infarction, as well as its utility in the context of the “working diagnosis” of MINOCA [1,19]. Dastidar et al. evaluated the prognostic role of CMR in a population of 388 patients presenting with MINOCA. In their study, CMR was able to identify a final diagnosis in 74% of patients and a CMR diagnosis of cardiomyopathy was one of the strongest predictors of mortality [8].

The prognosis of patients presenting with MINOCA depends strongly on the underlying cause and the use of its corresponding therapy [22,24]. In this context, our study underlines the paramount ability of CMR to guide the specific medical therapy for each definite diagnosis, since in our population it helped to adopt a different therapy regimen in 22% of patients.

In conclusion, as already stated in the American Heart Association (AHA) and the European Society of Cardiology guidelines for the management of acute coronary syndromes in patients presenting without persistent ST-segment elevation, MINOCA should be considered a working diagnosis, and the etiology pursued, excluding Takotsubo and myocarditis [1,25]. In this context, CMR imaging can robustly modify the therapy regimen and stratify the prognosis of ischemic patients hospitalized with a MINOCA diagnosis.

## 5. Study limitations

The single center design of this study is the principal limitation of the present investigation. Secondly, we adjusted for potential confounders in our analysis, but we cannot exclude residual confounding.

We only considered patients which underwent CMR, whereas it is possible that the inclusion of patients with suspected MINOCA who did not receive CMR could have biased our results; however, the purpose of the study was to analyze the CMR features of patients with the initial working diagnosis of MINOCA, and in our center the vast majority of these patients underwent CMR, although this may not be the routine approach in other hospitals. Moreover, coronary angiographies were not conducted utilizing the new intravascular diagnostic techniques, such as OCT or IVUS.

Finally, in treating ischemic MINOCA, it still largely debated whether ACE-Is or betablockers, in addition to antiplatelets agents or alone, can modify the patients’ prognosis; [22,23] unfortunately, the latter was not here investigated, but it could be the subject of future investigations.

## 6. Conclusions

In a contemporary cohort of MINOCA patients who underwent CMR, ischemic and non-ischemic patterns had distinct features and outcomes. CMR imaging can modify the therapy regimen and stratify the prognosis of ischemic patients hospitalized with a MINOCA diagnosis.

## Figures and Tables

**Table 1:** General characteristics according to ischemic and non-ischemic CMR patterns.

	Total	Non-Ischemic	Ischemic	
	*N* = 135	*n* = 80	*n* = 55	*p*-Value
Age (years)	49 ± 21	48 ± 21	55 ± 19	0.05
Female sex (*n*, %)	65 (48%)	17 (21%)	48 (87%)	**0.001**
**Comorbidities**				
Hypertension (*n*, %)	33 (24%)	14 (17%)	19 (34%)	**0.001**
Diabetes (*n*, %)	30 (22%)	11 (14%)	19 (34%)	**0.001**
Hypercholesterolemia (*n*, %)	33 (24%)	13 (16%)	20 (36%)	**0.001**
Smoker (*n*, %)	40 (30%)	21 (26%)	19 (34%)	0.058
Atrial fibrillation (*n*, %)	18 (13%)	6 (7%)	12 (22%)	**0.001**
ST-elevation (*n*, %)	55 (41%)	19 (24%)	36 (65%)	**0.001**
LVEF < 50% (*n*, %)	30 (22%)	16 (20%)	14 (25%)	0.058
**Laboratory**				
Troponins (ng/mL)	2.3 [1.0, 9.3]	1.9 [1.0, 7.7]	2.3 [1.3, 9.3]	0.72
CK-MB (ng/mL)	3.9 [2.9, 5.7]	3.3 [2.9, 5.1]	4.5 [3.5, 5.7]	0.55
Creatinine (g/dL)	0.9 ± 0.3	0.9 ± 0.2	1.0 ± 0.3	0.83
Hemoglobin (g/dL)	14.1 ± 1.7	14.1 ± 1.8	14.3 ± 1.6	0.67
C-reactive protein (mg/dL)	5.6 ± 6.0	5.5 ± 5.8	5.8 ± 6.8	0.87
**Therapy**				
NSAIDs (*n*, %)	110 (81%)	80 (100%)	30 (54%)	0.001
Aspirin (*n*, %)	102 (75%)	59 (74%)	43 (78%)	**0.022**
Clopidogrel (*n*, %)	42 (31%)	9 (11%)	33 (60%)	**0.001**
Ticagrelor (*n*, %)	24 (18%)	2 (2%)	22 (40%)	**0.001**
ACE-I (*n*, %)	84 (62%)	51 (64%)	33 (60%)	0.0004
ARBs (*n*, %)	65 (48%)	41 (51%)	24 (44%)	0.001
Statins (*n*, %)	75 (55%)	20 (25%)	55 (100%)	0.001
Beta-blockers (*n*, %)	22 (16%)	10 (12%)	12 (22%)	0.001

Legend: Cardiac magnetic resonance (CMR); left ventricle ejection fraction (LVEF); non-steroidal anti-inflammatory drugs (NSAIDs); angiotensin receptor blockers (ARBs); number (n); grams (g); deciliters (dL); milligrams (mg).

**Table 2:** General characteristics according to CMR ischemic patterns: focal and transmural.

	Total Ischemic	Focal	Transmural	
	*N* = 55	*n* = 32	*n* = 23	*p*-Value
Age (years)	55 ± 19	53.0 ± 19.3	40.1 ± 20.3	0.19
Female sex (*n*, %)	48 (87%)	29 (91%)	19 (83%)	0.16
**Comorbidities**				
Hypertension (*n*, %)	19 (34%)	2 (6%)	17 (74%)	**0.001**
Diabetes (*n*, %)	19 (34%)	1 (3%)	18 (78%)	**0.001**
Hypercholesterolemia (*n*, %)	20 (36%)	9 (28%)	11 (48%)	0.066
Smoker (*n*, %)	19 (34%)	10 (31%)	9 (39%)	0.26
Atrial fibrillation (*n*, %)	12 (22%)	2 (6%)	10 (43%)	**0.001**
ST-elevation (*n*, %)	36 (65%)	14 (44%)	22 (96%)	**0.047**
LVEF < 50% (*n*, %)	14 (25%)	3 (9%)	11 (48%)	**0.001**
**Laboratory**				
Troponins (ng/mL)	2.3 [1.3, 9.3]	1.3 [1.0, 2.3]	2.3 [1.3, 6.5]	**0.042**
CK-MB (ng/mL)	4.5 [3.5, 5.7]	3.3 [2.5, 4.5]	3.9 [2.9, 5.7]	**0.035**
Creatinine (g/dL)	1.0 ± 0.3	1.0 ± 0.3	1.0 ± 0.3	0.32
Hemoglobin	14.3 ± 1.6	14.4 ± 1.7	13.9 ± 1.8	0.71
C-reactive protein (mg/dL)	5.8 ± 6.8	4.6 ± 4.5	6.9 ± 8.8	0.68
**Therapy**				
NSAIDs (*n*, %)	30 (54%)	23 (72%)	7 (30%)	**0.001**
Aspirin (*n*, %)	43 (78%)	27 (84%)	16 (69%)	**0.036**
Clopidogrel (*n*, %)	33 (60%)	28 (87%)	5 (22%)	**0.001**
Ticagrelor (*n*, %)	22 (40%)	4 (12%)	18 (78%)	**0.001**
ACE-I (*n*, %)	33 (60%)	21 (66%)	12 (52%)	0.005
ARBs (*n*, %)	24 (44%)	5 (16%)	19 (83%)	0.003
Statins (*n*, %)	55 (100%)	32 (100%)	23 (100%)	0.25
Beta-blockers (*n*, %)	12 (22%)	9 (28%)	3 (13%)	**0.001**

Legend: Cardiac magnetic resonance (CMR); left ventricle ejection fraction (LVEF); non-steroidal anti-inflammatory drugs (NSAIDs); angiotensin receptor blockers (ARBs); number (n); grams (g); deciliters (dL); milligrams (mg).

**Table 3:** Independent predictors of cardiac-related rehospitalizations: the new occurrence of acute myocardial infarction (AMI), heart failure (HF), or arrhythmias.

	Haz. Ratio	[95% Confidence Interval]	*p*-Value
Age (per year)	1.96	1.94, 2.98	**0.001**
Female sex	1.47	0.52, 4.12	0.459
Hypertension	2.21	0.96, 5.06	0.060
Diabetes	0.43	0.17, 1.06	0.069
Hypercholesterolemia	1.14	1.04, 1.42	**<0.001**
ST-Elevation	6.26	3.19, 7.68	**0.001**
LVEF < 50%	3.60	1.45, 8.97	**0.006**
Transmural CMR Ischemic pattern	6.01	4.90, 7.53	**<0.001**

Legend: Cardiac magnetic resonance (CMR); left ventricle ejection fraction (LVEF).

## Data Availability

All data relevant to the study are included in the article.
